# Amyand's Hernia with Appendicitis: A Case Report and Integrative Review

**DOI:** 10.1155/2015/941039

**Published:** 2015-11-10

**Authors:** Jéssica Feitosa Cavalcante, Hermes Melo Teixeira Batista, Ivo Cavalcante Pita Neto, Jairo Fernandes Frutuoso, Woneska Rodrigues Pinheiro, Italla Maria Pinheiro Bezerra, Luiz Carlos de Abreu, Gylmara Bezerra de Menezes Silveira

**Affiliations:** ^1^Estácio FMJ Juazeiro do Norte, CE, Brazil; ^2^Laboratório de Delineamento e Escrita Científica, FMABC, Santo André, SP, Brazil; ^3^Hospital Regional do Cariri, Brazil

## Abstract

*Introduction.* Inguinal hernia is a common disorder with an estimated prevalence of 1.2% of the entire population and it is 12 times more common in males. *Objective.* To describe a case of appendix with signs of inflammation in the hernia sac, condition that is rare and difficult to diagnose, and to perform literature review, describing the most relevant aspects and the main controversies. *Method.* Report of a case and search in PubMed on June 1, 2015, using the terms “Appendix” [MeSH term] AND “hernia, inguinal” [MeSH term]. *Results.* The search resulted in 38 articles in total, and after deleting the articles that were not part of the inclusion criteria, there were 26 case reports remaining. *Discussion.* The search resulted in a total of 38 articles and after deleting the articles that were not part of the inclusion criteria, there were 26 case reports remaining. *Conclusion.* Amyand's hernia is a rare and difficult to diagnose condition, being commonly found occasionally in surgical procedures. It should be remembered in the presence of cases of incarcerated hernia, due to its possible complications if not diagnosed.

## 1. Introduction

Inguinal hernia is a frequent pathology with estimated prevalence of 1.2% of the population, with an incidence 12 times greater in the male gender. It affects both adults and children and the contents of the hernia sac may vary from epiplo, bowel, ovary, and appendix [1].

Amyand's hernia is characterized by the presence of the vermiform appendix in the inguinal hernia sac. It received its name because the first person to report the presence of perforated appendix in inguinal hernia was Claudius Amyand in 1736. The incidence is estimated at 1% of the inguinal hernias and its evolution to appendicitis reaches 0.1% [2].

Subsequently, a classification was established in three types: (A) intact appendix without signs of inflammation; (B) appendix with signs of inflammation; and (C) perforated appendix [3].

*Objective.* To describe a case of appendix with signs of inflammation in the hernia sac, that is a rare and difficult to diagnose condition, and to perform literature review, describing the most relevant aspects and the main controversies.

*Method.* Search on PubMed on June 1, 2015, using the terms “Appendix” [MeSH term] AND “hernia, inguinal” [MeSH term]. Review articles and articles that reported cases in children were excluded, plus the editorials and images. The inguinal hernia case reports in adults that contemplate the search terms and were published in English were included.

*Results.* The search resulted in 38 articles in total, and after deleting the articles that were not part of the inclusion criteria, there were 26 case reports remaining (Table 1).

## 2. Case Report

Male patient, 52 years old, coming from Serrita, Pernambuco, Brazil, sought medical assistance at Hospital Regional Inácio de Sá, in the city of Salgueiro in Pernambuco, Brazil, complaining of pain in the right inguinal region for three (3) days. At the physical examination a mass in the right inguinal region that increased with physical exertion has been found, irreducible to digital maneuvers with the presence of inflammatory signs. Additional tests were requested which demonstrated infectious white blood cells with 12,000 leukocytes, fasting glucose, urea, and creatinine within normal values. The patient underwent surgical treatment under the diagnosis of strangulated right inguinal hernia. Exploratory laparotomy was performed using an oblique incision in the right inguinal region. At the access of the inguinal canal, there was the existence of a hernia sac beside the deep epigastric vessels, which was part of the vermiform appendix. This was found with an increased size, signs of inflammation, infection, and necrosis of its apex, thus showing a necrotic appendicitis. Therefore, this case is the association of Amyand's hernia with an infectious and necrotic process of the appendix. An appendectomy and inguinal hernia surgery using the Shouldice technique were performed. The patient received ciprofloxacin and metronidazole upon the anesthetic induction and remained with this scheme during hospitalization, which lasted three days. Patient recovered well and was discharged on the third day after surgery with the prescription of the above-mentioned antibiotics for another 4 days at home. At the end there was a complete resolution of the case.

## 3. Discussion

Amyand's hernia is a rare condition where the vermiform appendix is part of the hernia content and may eventually evolve as appendicitis of difficult diagnosis. Until 1937 there were a total of 228 cases described in the literature [29].

**Figure 1:**
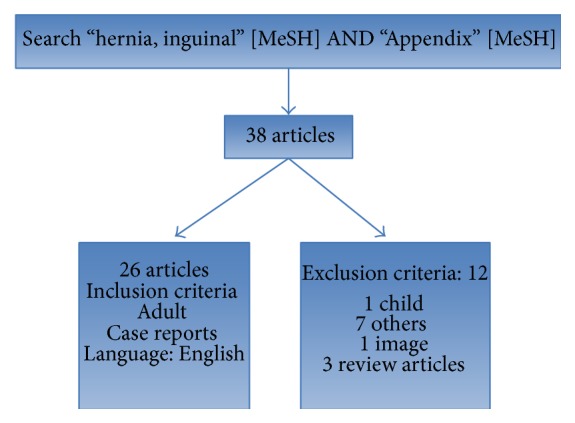
Flowchart from PubMed using the MeSH terms “hernia, inguinal” AND “Appendix.”

The search resulted in 38 articles in total, and after deleting the articles that were not part of the inclusion criteria, 26 case reports were remaining (Figure 1). Ultrasound and computed tomography are useful in the initial diagnosis [15, 30]. In the case reported, the diagnosis of Amyand's hernia with appendicitis was performed during the surgical procedure for the correction of incarcerated hernia.

**Figure 2:**
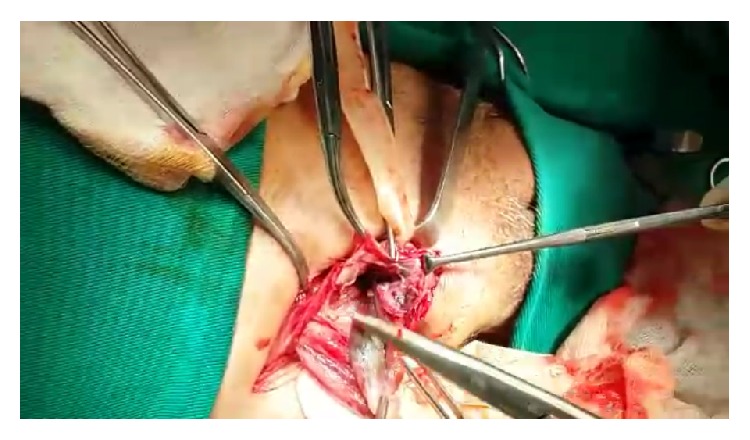
Hernia sac in the right inguinal region.

**Figure 3:**
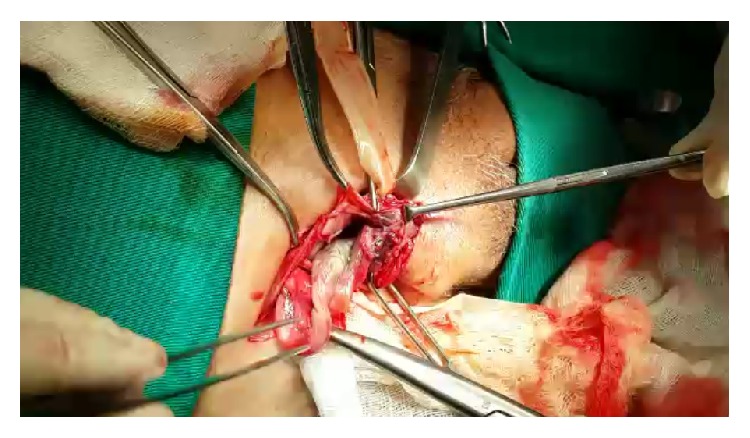
Cecal appendix with inflammatory signs composing the hernia sac.

**Figure 4:**
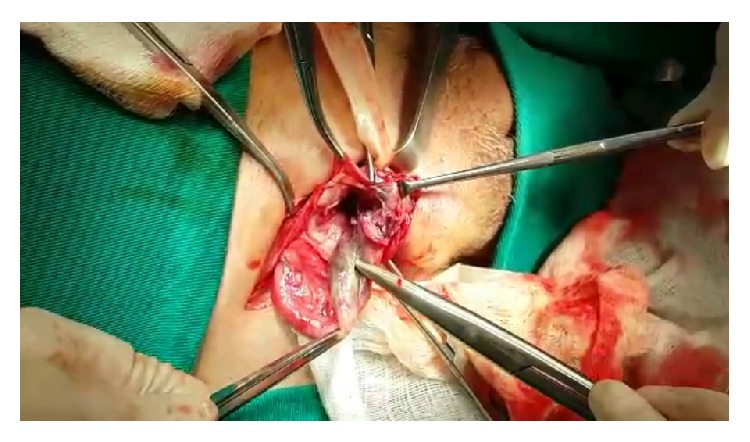
Necrosis of the apex of the appendix.

As the diagnosis is difficult at the beginning of the clinical condition, the presence of complications is common, often evolving to perforation and peritonitis, which along with older age are the main factors determining the prognosis [31]. In the case reported here, there was a late diagnosis and the appendix had already evolved to necrosis, without however having suffered perforation, as seen in Figures 2, 3, and 4.

The treatment comes down basically to the resolution of complications. When the appendicitis is found in Amyand's hernia, the performance of an appendectomy is virtually wise. However, some authors are reluctant when the appendix shows no signs of inflammation, while others suggest the realization of prophylactic appendectomy, citing the possibility of recurrence of hernia and the difficulty of later diagnosis of complications [32–35]. Findings on autopsy of Amyand's hernia in victims who have succumbed to other diseases show that this can remain asymptomatic throughout life [10].

Another common question is about the use of Marlex screen. Some suggest that its use increases the chances of surgical wound infection [36, 37]. However, when there are no signs of inflammation in the vermiform appendix and in the recurrence of hernia, most surgeons opt for screen use, although this implies longer hospital stay [38, 39]. In our case, an appendectomy was performed and hernia repair was performed using the Shouldice technique without using the screen because of risk of infection.

The differential diagnosis should be done with orchitis, Richter's hernia, and crural hernia. There are reports in the literature of Amyand's hernia associated with Richter's hernia evolving with appendicitis [40].

## 4. Conclusion

Amyand's hernia is a rare and difficult to diagnose condition, being commonly occasionally found in surgical procedure. It should be remembered in the presence of cases of incarcerated hernias, due to its possible complications if not diagnosed. Ultrasound or CT scan can help in the diagnosis and differentiate an incarcerated reducible inguinal hernia from Amyand's hernia evolving with appendicitis.

## Figures and Tables

**Table 1:** Articles selected from search in PubMed.

Author	Year	Sex	Age	Number of cases	Appendicitis
Amyand et al. [2]	1736	M	11	1	Grade C
Ceulemans et al. [3]	2014	M	70	1	Grade B
Dong et al. [4]	2014	M	63	1	Grade B
Hussain et al. [5]	2014	M	40	1	Grade B
Türkman et al. [6]	2013	M	73	1	Grade A
Lombardo and Pavone [7]	2013	M	47	1	Grade A
Maizlin et al. [8]	2007	M M F	86, 53, 83	3	Grade A, B, C
Ozkurt et al. [9]	2007	M	73	1	Grade B
Anagnostopoulou et al. [10]	2006	M	92	1	Grade A
Breitenstein et al. [11]	2005	F	81	1	Grade B
Junaid and Fawad [12]	2012	M	63	1	Grade A
Turanli et al. [13]	2011	M	54	1	Grade B
Mai [14]	2011	M	65	1	Grade C
Malayeri and Siegelman [15]	2011	M	84	1	Grade B
Coulier et al. [16]	2010	M	68	1	Grade B
Procter et al. [17]	2010	M	48	1	Grade C
Cunha et al. [18]	2009	M	22	1	Grade A
Yang et al. [19]	2009	M	70	4	Grade A
Johari et al. [20]	2009	M	73	1	Grade A
Doyle and McCowan [21]	2008	M	50	1	Grade B
Saggar et al. [22]	2004	M	77	1	Grade B
D'Alia et al. [23]	2003	M	84	1	Grade C
Franko et al. [24]	2002	M	73	1	Grade B
Hindle and Huang [25]	2002	M	65	1	Grade A
Fernando and Leelaratna [26]	2002	M	67	1	Grade B
Bamberger [27]	2001	M	50	1	Grade A
Goodwin and Ghilchik [28]	1998	M	53	1	Grade B
